# (Pyrrole-2,5-Diyl)-Bis(Nitronyl Nitroxide) and-Bis(Iminonitroxide): Specific Features of the Synthesis, Structure, and Magnetic Properties

**DOI:** 10.3390/molecules25071503

**Published:** 2020-03-26

**Authors:** Evgeny Tretyakov, Anastasia Tkacheva, Galina Romanenko, Artem Bogomyakov, Dmitri Stass, Alexander Maryasov, Ekaterina Zueva, Boris Trofimov, Victor Ovcharenko

**Affiliations:** 1 N. N. Vorozhtsov Institute of Organic Chemistry, Siberian Branch of Russian Academy of Sciences, 9 Ac. Lavrentiev Avenue, 630090 Novosibirsk, Russia; maryasov@nioch.nsc.ru; 2 International Tomography Center, Siberian Branch of Russian Academy of Sciences, 3a Institutskaya Str., 630090 Novosibirsk, Russia; tevtev@ngs.ru (A.T.); romanenk@tomo.nsc.ru (G.R.); bus@tomo.nsc.ru (A.B.); 3 V. V. Voevodsky Institute of Chemical Kinetics and Combustion, Siberian Branch of Russian Academy of Sciences, 3 Institutskaya Str., 630090 Novosibirsk, Russia; stass@kinetics.nsc.ru; 4 Novosibirsk State University, 2 Pirogova Str., 630090 Novosibirsk, Russia; 5 Kazan National Research Technological University, 68 Karl Marx Str., 420015 Kazan, Russia; zueva_ekaterina@mail.ru; 6 Kazan Federal University, 18 Kremlyovskaya Str., 420008 Kazan, Russia; 7 A. E. Favorsky Institute of Chemistry, Siberian Branch of Russian Academy of Sciences, 1 Favorsky Str., 664033 Irkutsk, Russia; boris_trofimov@irioch.irk.ru

**Keywords:** nitronyl nitroxides, iminonitroxides, diradicals, pyrrole derivatives, magnetochemistry, quantum-chemical calculations

## Abstract

In contrast to diradicals connected by alternant hydrocarbons, only a few studies have addressed diradicals connected by nonalternant hydrocarbons and their heteroatom derivatives. Here, the synthesis, structure, and magnetic properties of pyrrole-2,5-diyl–linked bis(nitronyl nitroxide) and bis(iminonitroxide) diradicals are described. The diradicals show characteristic electron spin resonance spectra in dilute glassy solutions, from which conclusions about the presence of distinct conformations, their symmetry, and interspin distance were made. X-ray diffraction analysis of the diradicals revealed that paramagnetic moieties lie in the plane of the pyrrole ring, because of the formation of an intramolecular hydrogen bond, O_NO_…H−N, with O…H distances of 2.15–2.23 Å. The N–O groups participating in the formation of H-bonds have greater bond lengths (~1.29 Å) as compared with nonparticipating groups (~1.27 Å). The nitronyl nitroxide and iminonitroxide diradicals showed an intramolecular antiferromagnetic interaction, with *J* = −77.3 and −22.2 cm^−1^, respectively (*H* = −2*J***S**_1_⋅**S**_2_).

## 1. Introduction

Delocalized high-spin organic compounds are actively studied with the aim of creating electrical conductors [1,2,3], magnetic switches [4,5,6,7,8,9], molecular spin-based quantum computers [10,11,12], and spintronic devices [13,14,15]. Delocalized diradicals, being the simplest type of high-spin organic molecules, can be categorized into non-Kekulé and Kekulé structures. Borden and Davidson have classified non-Kekulé structures into disjoint and nondisjoint alternant π-systems [16,17]. Although nondisjoint molecules contain singly occupied molecular orbitals (SOMOs) distributed over the whole molecule and have high-spin ground states, disjoint systems have SOMOs localized on separate molecular parts that may be connected either by the same sign of spin density or follow the alternant spin density pattern with S = 0. So far, different diradicals linked by alternant hydrocarbons have been actively researched theoretically [18,19,20,21,22,23] and experimentally [4,5,6,7,8,9]. Low- and high-spin states of such diradicals can be predicted via parity-based models, a disjoint–nondisjoint (coextensive) model, and a dynamic spin polarization model [18,19,20,21,22,23].

Against the background of actively investigated diradicals linked by alternant hydrocarbons, there are only a few reports about diradicals that are linked by nonalternant systems [24,25,26,27,28]. Nonbonding molecular orbitals (MOs) of such a diradical are denoted as disjoint diradicals (the two SOMOs are confined to distinct spatial regions), indicating that the triplet and singlet states are degenerate on the Hückel MO level, whereas the degeneracy tends to be disrupted via the inclusion of a configuration interaction to obtain singlet ground states [19]. On the other hand, due to rather small singlet–triplet energy gaps (|Δ*E*_ST_|) in a disjoint system, the ground state might depend on π-systems to which it is applied and on inclusion of heteroatoms in such a system.

**Figure 1:**
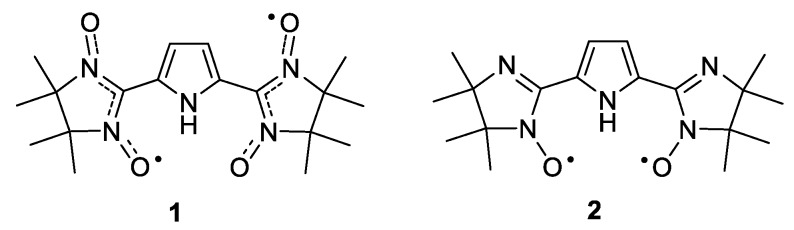
Chemical structure of bis(nitronyl nitroxide) **1** and bis(iminonitroxide) **2** diradicals.

In this study, we focused on pyrrole-2,5-diyl as a nonalternant hydrocarbon spacer and fully investigated bis(nitronyl nitroxide) **1** and bis(iminonitroxide) **2** diradicals shown in Figure 1. Here, we described specific features of the synthesis of diradicals **1** and **2**, their X-ray diffraction (XRD) structures, electron spin resonance (ESR) spectra, and the temperature dependence of magnetic-susceptibility values. After analysis of the obtained magnetic data, values of the parameters of the intramolecular exchange interaction were compared with those in other bis(nitronyl nitroxide) and bis(iminonitroxide) diradicals with a five-membered aromatic linker. 

## 2. Results and Discussion

**Scheme 1:**
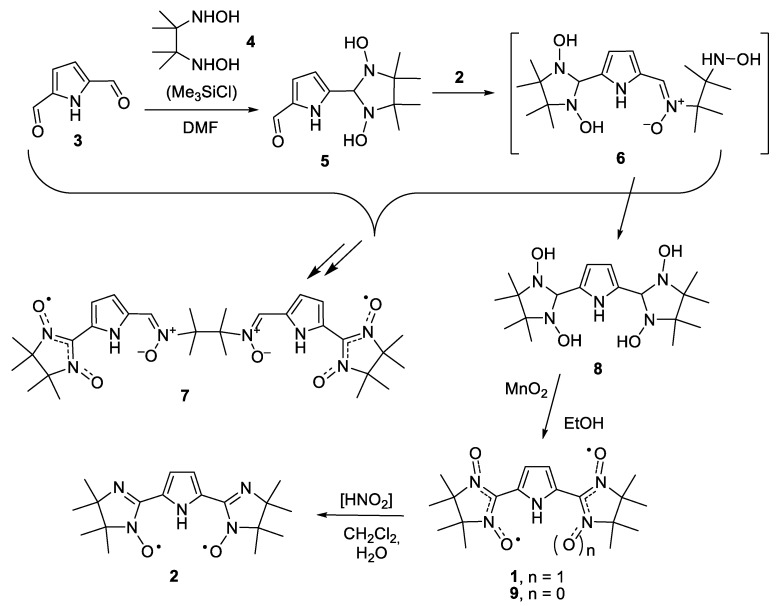
Preparation of diradicals **1**, **2**, **9**, and biradical **7**.

Diradicals **1** and **2** were prepared by interaction of dicarbaldehyde **3** with 2 equivalents of bis-hydroxylamine **4** with subsequent oxidation of the product. If the condensation reaction is carried out in dimethylformamide in the presence of 4 equivalents of Me_3_SiCl, then diadduct **8**, precipitating into a solid phase, contains virtually no impurities, and after oxidation, gives rise to **1** with the overall yield of 12% (Scheme 1). Reduction of **1** proceeds rapidly under standard conditions and leads to the formation of **2** with yields exceeding 80%. Good-quality crystals of **1** and **2** suitable for XRD analysis were obtained after recrystallization from a mixture of CH_2_Cl_2_ with heptane. 

Naturally, we attempted to carry out the condensation reaction without Me_3_SiCl too. Oxidation of the resultant precipitate gave a complex mixture of colored substances. From this mixture, by painstaking chromatographic separation, we finally isolated diradical **1** (16% yield), biradical **7** (20% yield), and trace amount of mixed diradical **9** (Scheme 1). The detection of substantial amounts of dinitrone biradical **7** among the reaction products may explain the lower yields of **1**. Apparently, at one of the stages of **1** precursor formation (namely, adduct **8** formation), an intermolecular process of condensation of hydroxylamine and aldehyde groups takes place, resulting in the formation of a dinitron bridge moiety between pyrrole rings and eventually—after oxidation—in the formation of biradical **7**. Fortunately, after recrystallization from the CH_2_Cl_2_–heptane mixture, the biradical **7** precipitated into a solid phase in the form of crystals suitable for XRD analysis, which revealed the structure of this complicated compound (see Supplementary Information; Figure S1). 

**Figure 2:**
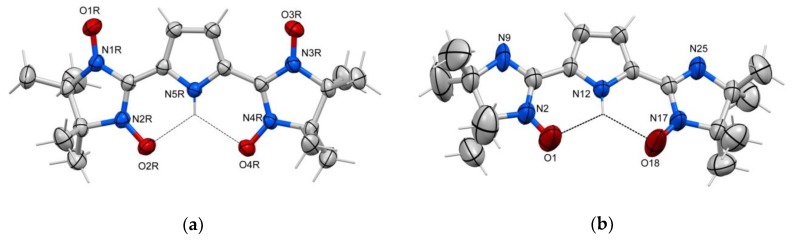

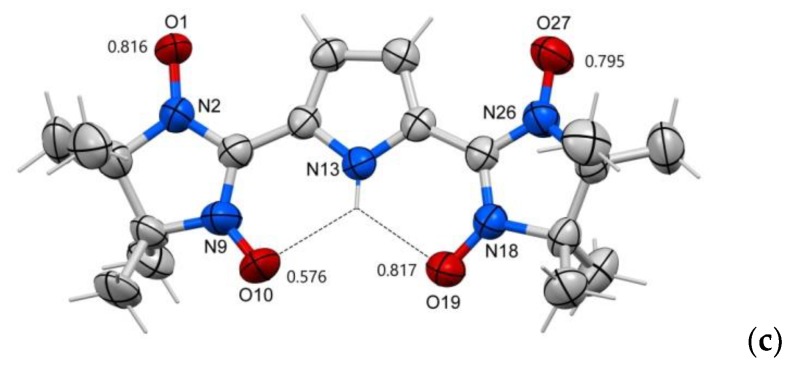
The structures of **1** (**a**), **2** (**b**), and **9** ((**c**); occupancy of positions of O atoms is indicated). The thermal ellipsoids are set at a 50% probability level.

The X-ray structural analysis of diradicals **1**, **2**, and **9** showed that both {CN_2_} parts of the imidazoline cycles are within the plane of the pyrrole ring because of the formation of an intramolecular hydrogen bond, O…H−N (Figure 2), with O…H distances of 2.15–2.23 Å. The N–O bond lengths in **1** for the O atoms participating in the formation of H-bonds (1.292(2) and 1.293(2) Å) are notably greater than the others (1.274(2) and 1.274(2) Å). In mixed diradical **9**, the O atoms are disordered (occupancy of positions is presented in Figure 2c). In **2** and **9**, the N–O bonds are substantially shorter and are within the ranges of 1.220(4)–1.242(4) and 1.243(3)–1.267(2) Å, respectively.

In all nitroxides, there are no short intermolecular contacts between nitroxide groups. In the crystal structure of **1**, the shortest intermolecular contacts with participation of nitroxide groups are five contacts between O atoms and H atoms of methyl groups (2.526–2.604 Å); in **2** there are pair contacts between O atoms and a C−H pyrrole ring (O…C 3.361 Å) to form ribbons; in **9** there are contacts between O atoms and H_CH3_ atoms lying in the range of 2.553–2.708 Å.

**Figure 3:**
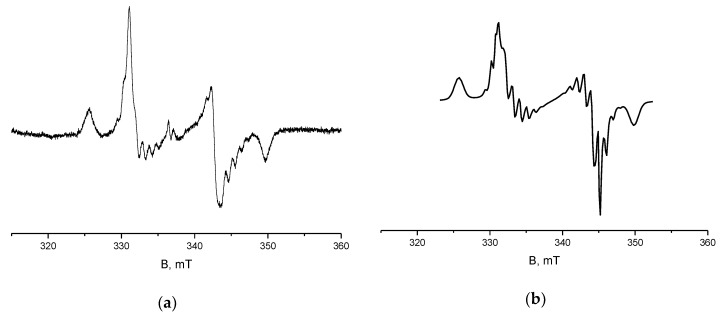
The electron spin resonance (ESR) spectrum of diradical **1** in a diluted glassy solution: experiment (**a**) and modeling (**b**).

**Figure 4:**
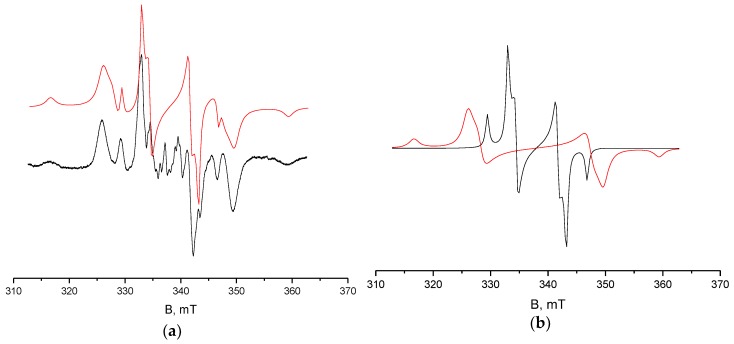
The ESR spectrum of diradical **2** in a diluted glassy solution: experiment and its modeling (**a**), and two components of the model spectrum (**b**).

**Figure 5:**
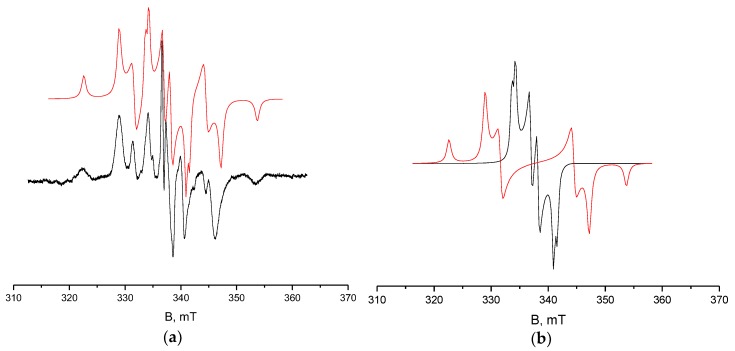
The ESR spectrum of diradical **9** in a diluted glassy solution: experiment and its modeling (**a**) and two components of the model spectrum (**b**).

Continuous-wave ESR spectra of diluted glassy solutions of **1**, **2**, and **9** are typical of diradicals with *S* = 1 and comprise a characteristic spectrum in the region 340 mT (g = 2) and a weak line in half-field. Figure 3, Figure 4 and Figure 5 show representative spectra of each diradical in the region g = 2 as well as the results of their modeling and interpretation. 

The spectra were modeled within the framework of second-order perturbation theory to account for a rather noticeable asymmetry, using the expressions from ref. [29] and spin Hamiltonian with parametrization: $\overset{⌢}{H}=g\beta B_{0}{\overset{⌢}{S}}_{z}^{}+D[{\overset{⌢}{S}}_{z}^{2}-2/3]+E[{\overset{⌢}{S}}_{x}^{2}-{\overset{⌢}{S}}_{y}^{2}].$ The modeling yielded parameters *D* and *E*, and the value of *D* provided an estimate of the distance between the effective point dipoles as *D* = 3/2(*g*^2^*β*^2^*r*^−3^), while the *E*/*D* ratio gave an indirect measure of the degree of coplanarity of the two radical moieties in the molecule. 

Although for **2** and **9**, the spectrum is a superposition of spectra of two distinct diradicals with well-defined and quite different geometry (which likely correspond to different relative positioning of the two radical moieties), a single conformation is sufficient to reproduce the spectrum of **1**. Furthermore, the symmetry of this molecule is so high that the spectrum contains explicitly displayed hyperfine interactions with four equivalent nitrogen atoms, which could be reproduced in modeling by augmenting the Hamiltonian with the relevant $\vec{S}A\vec{I}$ terms. In the experimental spectra of **1**, the relative intensity of the doublet at the spectrum center decreases with decreasing diradical concentration (see Supplementary Materials); this finding means that the doublet belongs to the compact phases forming at the surface of cracks that developed in the glass; this doublet was ignored in the modeling. Parameters *D* and *E*/*D* were found to be 12.04 mT and 0.0664, and the estimated distance between the point dipoles is 0.61 nm. The hyperfine tensor of the four assumed equivalent nitrogen atoms in the system of a zero field splitting tensor turned out to be diagonal with eigenvalues (0.3, 0.2, and 0.97) mT, also indicating high symmetry of this diradical in a diluted glassy solution. Figure 3 shows a comparison of the experimental and simulated spectra of diradical **1**. 

The spectrum of **2** was modeled as a superposition of two spectra (Figure 4); the effects of hyperfine interactions and g-tensor anisotropy were not included. As in the case of **1**, in the experimental spectra, relative intensity of the doublet at the spectrum center decreases with decreasing diradical concentration (see Supplementary Materials), and the doublet is attributed to deposits of compact phases forming due to poor solubility in glass. Both model spectra show slight asymmetry changing the shape of the inner lines of the spectrum. The parameters of the broad component were found next: *D* = 21.35 mT and *E*/*D* = 0.0351; the estimated distance between point dipoles is 0.50 nm. The corresponding values for the narrow component are *D* = 8.69 mT, *E*/*D* = 0.0576, and 0.68 nm, respectively. 

The spectrum of diradical **9** was also modeled as a superposition of two spectra (Figure 5) ignoring the effects of hyperfine interactions and g-tensor anisotropy. The obtained parameters of the broad component are *D* = 15.56 mT and *E*/*D* = 0.058. The symmetry of the corresponding conformation of the diradical slightly differs from axial, and a noticeable splitting of central lines appears. The estimated distance between point dipoles is 0.56 nm. The parameters of the narrow component are *D* = 3.98 mT and *E*/*D* = 0.226. The unusually high *E*/*D* ratio (its maximum possible value is ^1^/_3_) indicates that the symmetry of the corresponding conformation of the diradical is far from axial; a resolved doublet structure appears in the center of the spectrum. In contrast to the other two diradicals, in the experimental spectra of **9**, this central doublet did not depend on the concentration of the diradical (see Supplementary Materials) and was therefore attributed to the diradical itself; compact-phase formation was not observed due to better solubility of the nonplanar compound. The estimated distance between point dipoles is 0.88 nm. 

**Figure 6:**
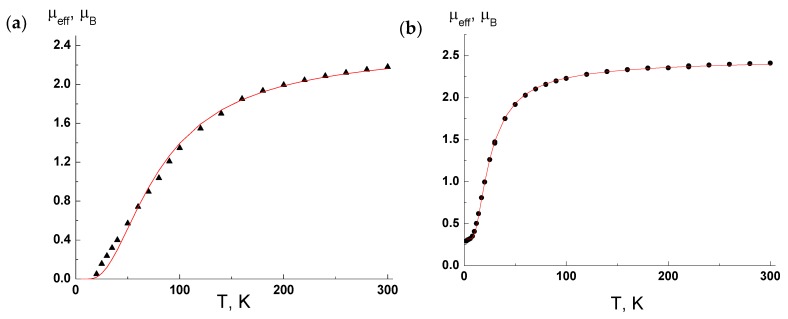
The μ_eff_(*T*) dependencies for **1** (**a**) and **2** (**b**). Solid curves are theoretical.

Temperature dependencies of µ_eff_ for **1** and **2** are depicted in Figure 6. The µ_eff_ values at 300 K are 2.18 and 2.41 µ_B_, respectively, for **1** and **2** and are in good agreement with the theoretical value of 2.45 µ_B_ for two uncoupled spins with S = 1/2 at g = 2. A decrease in µ_eff_ with decreasing temperature means the predominance of antiferromagnetic coupling between nitroxide spins. Analysis of the µ_eff_(*T*) dependencies was performed using a model of an exchange-coupled dimer (spin Hamiltonian *H* = −2*J***S**_1_⋅**S**_2_). The best-fit values of the g-factor and exchange coupling parameter *J* are 1.99 (±0.02) and −77.3 (±1.2) cm^−^^1^ for **1** and 2.01 (±0.01) and −22.2 (±0.1) cm^−^^1^ for **2**, respectively. Magnetochemical analysis is in agreement with the results of theoretical studies by S. Datta [30,31], according to which antiferromagnetic coupling (*J* = −161 and −164 cm^−^^1^) should be expected in the **1** molecule. 

The results of quantum-chemical calculations obtained via a spin-projected broken-symmetry density functional theory (BS-DFT) approach are qualitatively consistent with the experimental data: *J* values computed for **1** and **2** at their crystallographically determined geometries are −288 and −88 cm^−1^, respectively. Note also that the value of *J* = −169 cm^−1^ computed using the optimized geometry of **1** matches the quantum-chemical value obtained earlier [11] within the same computational framework. 

The spin-projected BS-DFT schemes provide reliable information about the sign and relative importance of intra- and intermolecular exchange pathways. Indeed, our calculations reproduce the sign of the isotropic exchange parameter and the nature of its variation within a given series of diradicals. Nonetheless, the energy of antiferromagnetic exchange coupling is substantially overestimated at the BS-DFT level. More accurate quantum-chemical *J* values can be obtained using multiconfigurational wave function-based approaches. 

Table 1 summarizes the results obtained at the level of complete active-space multiconfigurational self-consistent field CASSCF(*n*,*m*)/*n*-electron valence state second-order perturbation theory (NEVPT2). The lowest singlet and triplet electronic states were calculated using the state-specific (SS) and state-average (SA) CASSCF(*n*,*m*)/NEVPT2 procedures, i.e., the SS- and SA-CASSCF(*n*,*m*) procedures were employed to obtain the MOs to be used at the NEVPT2 step. In the SS case, CASSCF(*n*,*m*) MOs were obtained separately for each state. In the SA case, the same (averaged) CASSCF(*n*,*m*) MOs were used for both states. The complete active space CAS(*n*,*m*) SOMOs obtained for **1** and **2** are shown in Figures S2 and S3, respectively. The minimal CAS consists of singly occupied MOs. As presented in Table 1, the use of the minimal CAS does not yield a satisfactory description of the magnetic exchange coupling. Enlarging the minimal CAS to CAS(10,10) enhances the antiferromagnetic interaction, with the only exception observed for **1** (see the NEVPT2 results obtained with SA-MOs). Further extension of the active space shifts the isotropic magnetic exchange parameter toward less negative values. The inclusion of all valence π-MOs gives rise to CAS(full valence π): CAS(20,15) for **1** and CAS(16,13) for **2**. At the CASSCF(full valence π)/NEVPT2 level, *J* values obtained with SS- and SA-MOs become comparable, and quantitative agreement with the experimental data is achieved, especially in the SA case (*J*_calc_ = −96 cm^−1^ for **1** and −31 cm^−1^ for **2**). 

The experimental and theoretical data indicate that in studied diradicals **1** and **2**, the pyrrole-2,5-diyl linker is a relatively effective coupling unit providing an antiferromagnetic exchange interaction between paramagnetic parts. In this regard, a question arises: how does the exchange interaction in nitronyl nitroxide and iminonitroxide diradicals depend on the structure of the five-membered aromatic linker? All available experimental data on the magnetic properties of diradicals of this type are compiled in Table 2. 

**Figure 7:**
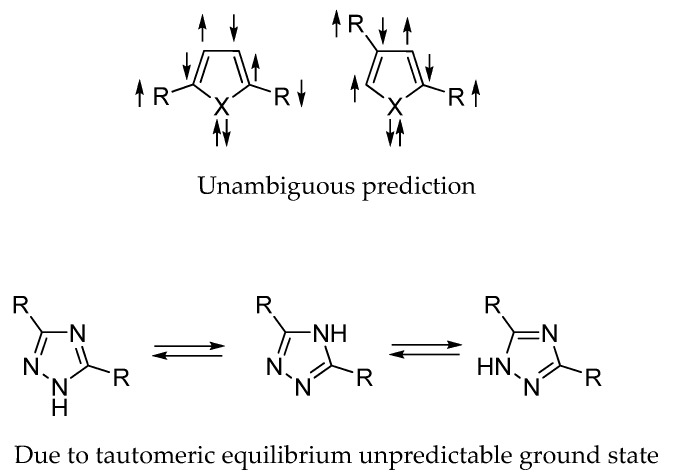
Prediction of the sign of the exchange coupling (R: nitronyl nitroxide or iminonitroxide moieties).

First, one can see that in almost all diradicals, the angle between planes of an aromatic cycle and paramagnetic parts does not exceed 11°. Second, in diradicals with the same coupler, the energy of exchange interaction is approximately threefold greater in bis(nitronyl nitroxides) than in bis(iminonitroxides). Third (the most important observation), five-membered hetarene-2,5-diyl couplers provide an antiferromagnetic exchange interaction; at the same time, diradicals with hetar-2,4-diyl or azulene-1,3-diyl couplers have a triplet ground state. This means that in the range of five-membered heteroaromatic couplers, the 2,5-diyl and 2,4-diyl species belong to *p*-phenylene and *m*-phenylene couplers, respectively. In other words, the valence bond theoretical picture of the spin polarization via carbon π-orbitals may be identical to that in alternant hydrocarbons, but a path via the heteroatom should involve a superexchange of a lone pair of electrons (Figure 7) [32]. The 1,2,4-triazole coupler is a special case because it can exist in different tautomeric forms belonging to 2,4-diyl or 2,5-diyl linkers. This is probably the reason why the very weak antiferromagnetic interaction is observed in the 1,2,4-triazole-coupled diradical [32]. 

## 3. Conclusions

Thus, in this study, nitronyl- and imino-nitroxyl diradicals with the pyrrole-2,5-diyl coupler were synthesized and thoroughly investigated. In these diradicals, the paramagnetic parts lie in the plane of the pyrrole ring because of the formation of an intramolecular H-bond: O…H−N. ESR spectroscopy gave insight into the structures of the diradicals in glassy solutions and provided a quantitative estimate of spin–spin distances. An additional useful outcome of the ESR analysis, covered in more detail in Supplementary Materials, was the demonstrated exceptional stability of the diradicals. In the studied diradicals, the pyrrole-2,5-diyl coupler corresponds to the *p*-phenylene parity, which explains the observed intramolecular exchange interactions of antiferromagnetic character.

## 4. Experimental Part

### 4.1. Reagents and General Methods

1*H*-Pyrrole-2,5-dicarbaldehyde [34] and 2,3-bis-hydroxylamino-2,3-dimethylbutane [35] were synthesized by known methods. Commercially available reagents and solvents were used without further purification. Thin-layer chromatography was performed on Silica Gel 60 F_254_ plates with a fixed adsorbent layer on aluminum foil. For column chromatography, silica gel with grain size 0.063–0.200 mm (Merck) was employed. Elemental analysis was conducted on a Euro EA 3000 microanalyzer. Infrared (IR) spectra of samples in KBr were recorded on a Bruker Vector-22 spectrophotometer. Magnetochemical measurements were carried out with SQUID magnetometer Quantum Design MPMS*XL* in the temperature range 2–300 K. 

### 4.2. Procedures for Preparation of 2,2′-(1H-pyrrole-2,5-diyl)-bis(4,4,5,5-tetramethyl-4,5-dihydro-1H-imidazole-3-oxide-1-oxyl) (1)

*Method 1.* To a stirred mixture of 2,3-bis-hydroxylamino-2,3-dimethylbutane **4** (2.40 g, 0.0162 mol), 1*H*-pyrrole-2,5-dicarbaldehyde **3** (1.00 g, 0.00812 mol), and dimethylformamide (30 mL) in an inert atmosphere, we added trimethylchlorosilane (3.53 g, 4.13 mL, 0.0325 mol). The reaction mixture was stirred in the inert atmosphere for 7 days, and the resulting swamp-green precipitate was filtered off and air dried (3.40 g). The obtained product was dissolved in ethanol (80 mL), and next, MnO_2_ (20.00 g, 0.230 mol) and NaF (1.365 g, 0.0325 mol) were added. The mixture was stirred for 20 h and filtered, and the resultant solution was evaporated in vacuum. The residue was crushed in 10 mL of toluene, causing its crystallization. The crystalline product was separated on a filter and purified by column chromatography on silica gel (eluent: AcOEt). Yield 0.373 g (12%), dark-green crystalline powder, *R_f_* (SiO_2_, EtOAc) 0.52. IR spectrum, *ν*/cm^−1^: 3308, 2985, 2940, 1633, 1594, 1556, 1536, 1455, 1412, 1381, 1372, 1341, 1295, 1217, 1195, 1170, 1141, 1095, 1041, 1011, 989, 910, 880, 864, 801, 748, 645, 587, 543, 484, 463. Found, %: C, 57.4; H, 7.1; N, 18.8. Calculated for C_18_H_27_N_5_O_4_, %: C, 57.3; H, 7.2; N, 18.6. 

*Method 2.* To a mixture of 2,3-bis-hydroxylamino-2,3-dimethylbutane **4** (2.40 g, 0.0162 mol) and 1*H*-pyrrole-2,5-dicarbaldehyde **3** (1.00 g, 0.00812 mol), we added dimethylformamide (30 mL). The reaction mixture was stirred in an inert atmosphere for 30 days and was evaporated in vacuum. The residue was crushed in diethyl ether (20 mL), and the suspension was kept at −10 °C for 20 h. After separation of the diethyl ether solution, an oily residue (0.320 g) was concentrated in vacuum for 2 h and dissolved in EtOH (25 mL). The obtained solution was mixed with MnO_2_ (3.20 g, 0.0368 mol), and the mixture was stirred for 1.5 h, filtered, and evaporated. The residue was purified by column chromatography on silica gel (eluents: CHCl_3_ and then AcOEt). The diethyl ether mother liquor was concentrated in vacuum, and the residue was dissolved in EtOH (30 mL). To this solution, MnO_2_ (6.00 g, 0.0690 mol) was added, and the mixture was stirred for 2 h at room temperature, filtered, and evaporated in vacuum. The residue was also purified by column chromatography on silica gel. The total yield of the product (**1**) was 0.483 g (16%). In addition to **1**, during the chromatographic purification, we isolated diradicals **7** and **9** with yields of 0.503 g (20%) and 0.010 g (0.3%), respectively.

(*N*^2^*Z*,*N*^3^*Z*)-*N*^2^,*N*^3^-Bis((5-(1-oxyl-4,4,5,5-tetramethyl-3-oxido-4,5-dihydro-1*H*-imidazol-2-yl)-1*H*-pyrrol-2-yl)methylene)-2,3-dimethylbutane-2,3-diamine dioxide (7). Needlelike dark-green crystals, *R_f_* (SiO_2_, AcOEt) 0.23. IR spectrum, *ν*/cm^−1^: 3455, 3102, 2988, 2943, 1638, 1593, 1566, 1499, 1453, 1425, 1382, 1371, 1357, 1297, 1205, 1172, 1129, 1074, 1042, 1003, 988, 887, 867, 808, 740, 675, 610, 592, 541, and 466.

2-(5-(1-Oxyl-4,4,5,5-tetramethyl-4,5-dihydro-1*H*-imidazol-2-yl)-1*H*-pyrrol-2-yl)-4,4,5,5-tetramethyl-4,5-dihydro-1*H*-imidazole-3-oxide-1-oxyl (9). Dark purple crystals, *R_f_* (SiO_2_, AcOEt) 0.60. IR spectrum, *ν*/cm^−1^: 3341, 2980, 2937, 1752, 1602, 1491, 1439, 1422, 1374, 1352, 1310, 1263, 1226, 1198, 1173, 1135, 1102, 1037, 1008, 989, 867, 801, 714, 688, 617, 592, 560, 540, 465, and 441. 

### 4.3. Synthesis of 2,2′-(1H-pyrrole-2,5-diyl)bis(4,4,5,5-tetramethyl-4,5-dihydro-1H-imidazole-1-oxyl) (2)

To a solution of 2,2′-(1*H*-pyrrole-2,5-diyl)-bis(4,5-dihydro-1*H*-imidazole-3-oxide-1-oxyl) (0.200 g, 0.529 mmol) and NaNO_2_ (0.110 g, 1.587 mmol) in CHCl_3_ (15 mL) and H_2_O (0.7 mL), AcOH (0.095 g, 1.587 mmol) was added. The reaction mixture was stirred for 30 min at room temperature, and then NaHCO_3_ (0.160 g) was added, followed by MnO_2_ (0.010 g, 0.115 mmol) in 5 min. The mixture was stirred for 5 min, dried with Na_2_SO_4_, and filtered. The residue was purified by column chromatography on silica gel. Yield 0.152 g (83%), crimson crystals, *R_f_* (SiO_2_, AcOEt) 0.61. IR spectrum, *ν*/cm^−1^: 3455, 3376, 3133, 2977, 2931, 2907, 2836, 1586, 1521, 1447, 1398, 1372, 1307, 1282, 1256, 1201, 1182, 1134, 1094, 1052, 1009, 990, 945, 876, 830, 773, 728, 711, 684, 604, 592, 559. Found, %: C, 62.6; H, 7.9; N, 20.3. Calculated for C_18_H_27_N_5_O_2_, %: C, 62.2; H, 7.6; N, 20.1.

### 4.4. X-Band ESR Measurements

Continuous-wave ESR spectra of diradicals **1**, **2**, and **9** were recorded on a Bruker EMX spectrometer in glassy *o*-terphenyl at 200 K (unless stated otherwise) and modeled in Wolfram Mathematica, in accordance with a previously developed protocol [36]. For this analysis, a crystal of a compound under study was placed in a 1.5 mL Eppendorf microfuge tube, *o*-terphenyl (T_m_ = 343 K) was added as a powder, and the mixture was melted on a water bath with stirring to produce a homogeneous melt. After cooling down and solidification, the mixture was ground, and a small amount of this diradical prediluted in *o*-terphenyl was placed into a standard quartz ESR tube with a 3-mm inner diameter. Neat *o*-terphenyl powder was then added into the tube, and the mixture was again melted on the water bath with stirring by a glass capillary to obtain a homogeneous melt. After that, the tube was sealed with parafilm, rapidly taken out of the bath, wiped dry, and placed in liquid nitrogen, resulting in a glassy sample after this shock freezing. After some time in liquid nitrogen, the sample was transferred to the resonator of the spectrometer equipped with a standard temperature controller preset to 200 K and was left for some time to warm up to 200 K before the measurements.

### 4.5. Crystallographic Analysis

XRD analyses were conducted on Bruker AXS diffractometers: SMART APEX II and Apex Duo (absorption was registered by the SADABS software, version 2.10). Structures were solved by direct methods and refined by the full-matrix least-squares method with anisotropic approximation for all nonhydrogen atoms. H atom positions were partially calculated geometrically and refined via the “riding” model. All the computations for the determination and refinement of the structures were carried out using the Bruker Shelxtl software suite (Version 6.14) and SHELX2016/6 [37]. Crystallographic characteristics of the studied compounds and experimental details are listed in Table S1. Complete information about the structures can be found at the Cambridge Crystallographic Data Centre (CCDC 1965209–1965211; deposit@ccdc.cam.ac.uk; http://www.ccdc.cam.ac.uk/data_request/cif).

### 4.6. Magnetic Measurements

Magnetic susceptibility measurements were taken on SQUID magnetometer Quantum Design MPMS*XL* in the temperature range of 2–300 K at a magnetic field strength of 5 kOe. Paramagnetic components of magnetic susceptibility were determined by taking into account the diamagnetic contribution evaluated through Pascal constants. Effective magnetic moment was calculated according to the formula μ_eff_ = [3*k*χ*T*/(*N*_A_μ_B_^2^)]^1/2^, where *N*_A_, μ_B_, and *k* are the Avogadro number, Bohr magneton, and Boltzmann constant, respectively.

### 4.7. Quantum-Chemical Calculations

Quantum-chemical calculations were performed at the X-ray geometries. Computations at the BS-DFT level were done via the Gaussian 09 package [38], whereas calculations at the CASSCF(*n*,*m*)/NEVPT2 level were done using the ORCA 4.0 package [39].

The high-spin and broken-symmetry single determinants were calculated using the B3LYP functional in conjunction with the 6-311+G(d,p) basis set. The internal stability of all SCF solutions was confirmed by stability analysis. The isotropic exchange parameter was estimated in accordance with the spin-projected scheme proposed by Yamaguchi and coworkers [40].

In the CASSCF(*n*,*m*)/NEVPT2 calculations, the def2-TZVP basis set was utilized. In the CASSCF(*n*,*m*)/NEVPT2 method, the reference wave function is calculated at the CASSCF(*n*,*m*) level, and then the remaining electron correlation is included via the multiconfigurational NEVPT2 approach. The SA- and SS-CASSCF(*n*,*m*) procedures were employed to obtain the MOs to be used at the NEVPT2 step. The SA-CASSCF(*n*,*m*) MOs were obtained from the average of the lowest singlet and triplet states with equal weights. The restricted open-shell Hartree-Fock/second-order Moller-Plesset perturbation theory natural MOs optimized for the triplet state served as an initial guess. The SS-CASSCF(*n*,*m*) MOs were obtained starting from the corresponding SA-CASSCF(*n*,*m*) MOs. The CASSCF energy and orbital gradient convergence tolerances were set to 10^−9^ and 10^−6^, respectively. The RI approximation was applied throughout. The *J* values shown in Table 1 were computed within the strongly contracted NEVPT2 framework. The use of the fully internally contracted NEVPT2 formalism yielded similar results. 

## Figures and Tables

**Table 1:** *J* values (in cm^−1^) computed for **1** and **2** at the complete active-space multiconfigurational self-consistent field CASSCF(*n*,*m*)/*n*-electron valence state second-order perturbation theory (NEVPT2) level.

	SS-MOs		SA-MOs	
(*n*,*m*) *^a^*	CASSCF	NEVPT2	CASSCF	NEVPT2
(*a*) **1**				
(2,2)	0.2	−140	0.2	−140
(6,6)	−61	−173	−61	−55
(10,10)	−135	−188	−135	−106
(16,13)	−124	−138	−124	−93
(20,15)	−116	−114	−116	−96
(*b*) **2**				
(2,2)	1.2	2.8	1.2	−3.5
(6,6)	−21	−35	−21	−22
(10,10)	−48	−58	−48	−41
(16,13)	−47	−39	−47	−31

*^a^* (*n*,*m*) is active-space size (*n* electrons in *m* orbitals). SS—state-specific, SA—state-average, MO—molecular orbital.

**Table 2:** Structures of nonalternant linkers, angles between planes of linkers and paramagnetic moieties, and experimental *J* values of intramolecular exchange interactions in diradicals.

Paramagnetic Parts	Linkers	Angles, °	J Value, K ^a^	Reference
NN ^b^	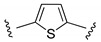	7.8, 10.5	−115	[32]
NN	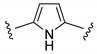	0	−111	This work
NN	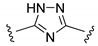	–	−1.6	[33]
NN	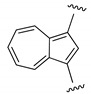	~43	10	[28]
NN	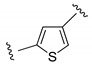		40	[32]
IN	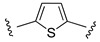	–	−30	[32]
IN	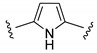	0	−32	This work
IN	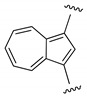	~5, ~11	0–1	[28]
IN	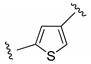	–	16	[32]

*^a^* The magnetic coupling constants quoted correspond to a Hamiltonian of the form *H* = −2*J***S**_1_⋅**S**_2_. *^b^* NN and IN denote nitronyl nitroxide and iminonitroxide moieties, respectively.

## Supplementary Materials

The following are available online, Table S1: Crystal data and details of experiments for diradicals **1**, **2**, **9**, and biradical **7**·CH_2_Cl_2_, Figure S1: Molecular structure of **7**, Figure S2: CAS(20,15) MOs obtained for **1**, Figure S3: CAS(16,13) MOs obtained for **2**, Figures S4–S9: ESR spectra of diradical **1** in *o*-terphenyl, Figures S10–S15: ESR spectra of diradical **9** in *o*-terphenyl, Figures S16–S20: ESR spectra of **2** in *o*-terphenyl.
