# Supplementary material for: (Pyrrole-2,5-Diyl)-Bis(Nitronyl Nitroxide) and-Bis(Iminonitroxide): Specific Features of the Synthesis, Structure, and Magnetic Properties
Source: Molecules. 2020 Mar 26;25(7):1503. doi: 10.3390/molecules25071503 (PMC7180855; doi:10.3390/molecules25071503)
Supplement: Supplementary file 1 [file molecules-25-01503-s001.pdf]

Supporting Information

(Pyrrole-2,5-Diyl)-Bis(Nitronyl Nitroxide) and-Bis(Iminonitroxide): Specific Features of the Synthesis, Structure, and Magnetic Properties

Evgeny Tretyakov [1,*], Anastasia Tkacheva [2], Galina Romanenko [2], Artem Bogomyakov [2], Dmitri Stass [3,4], Alexander Maryasov [1], Ekaterina Zueva [5,6], Boris Trofimov [7] and Victor Ovcharenko [2,*]

[1] N. N. Vorozhtsov Institute of Organic Chemistry, Siberian Branch of Russian Academy of Sciences, 9 Ac. Lavrentiev Avenue, Novosibirsk 630090, Russia; maryasov@nioch.nsc.ru

[2] International Tomography Center, Siberian Branch of Russian Academy of Sciences, 3a Institutskaya Str., Novosibirsk 630090, Russia; tevtev@ngs.ru (A.T.); romanenk@tomo.nsc.ru (G.R.); bus@tomo.nsc.ru (A.B.)

[3] V. V. Voevodsky Institute of Chemical Kinetics and Combustion, Siberian Branch of Russian Academy of Sciences, 3 Institutskaya Str., Novosibirsk 630090, Russia; stass@kinetics.nsc.ru

[4] Novosibirsk State University, 2 Pirogova Str., Novosibirsk 630090, Russia;

[5] Kazan National Research Technological University, 68 Karl Marx Str., 420015 Kazan, Russia; zueva_ekaterina@mail.ru

[6] Kazan Federal University, 18 Kremlyovskaya Str., 420008 Kazan, Russia;

[7] A. E. Favorsky Institute of Chemistry, Siberian Branch of Russian Academy of Sciences, 1 Favorsky Str., Irkutsk 664033, Russia; boris_trofimov@irioch.irk.ru

* Correspondence: tretyakov@nioch.nsc.ru (E.T.); ovchar@tomo.nsc.ru (V.O.)

Contents:

XRD analysis……………………………………………………………………..S2

Quantum-chemical calculations……………………………………………………..S3

CW ESR Spectroscopy………………………………………………………………S7

XRD analysis

Table S1. Crystal data and details of experiments for diradicals **1**, **2**, **9** and biradical **7**$\cdot CH_2Cl_2$.

	**1**	**2**	**9**	**7**$\cdot CH_2Cl_2$
FW	377.45	345.44	361.44	656.20
T, K	296	240	296	296
Sp. gr., *Z*	$P2_1/n$, 4	*Pnma*, 4	$P2_1/n$, 4	$Pna2_1$, 4
a, Å	11.2152(5)	28.057(5)	12.2174(18)	13.325(3)
b, Å	11.2380(6)	9.659(2)	13.139(2)	29.840(7)
c, Å	15.8538(7)	7.1211(18)	12.872(2)	9.149(2)
β, °	94.450(2)		104.686(11)	
V, Å^3	1992.13(16)	1929.7(7)	1998.8(5)	3637.6(15)
D_c, g/cm^3	1.258	1.189	1.201	1.198
θ_{max}, °	65.00	27.961	64.325	28.00
I_{hkl} coll/uniq	9163 / 3321	16725 / 2439	19037 / 3303	19582 / 7948
R_{int}	0.0954	0.1035	0.1010	0.1312
I_{hkl} uniq (I>2σ_I) / N	2700 / 245	827 / 143	2369 / 294	2072 / 425
Goof	1.076	0.938	1.034	0.683
$R1$ / $wR2$ (I>2σ_I)	0.0428 / 0.1176	0.0817 / 0.2038	0.0529 / 0.1507	0.0562 / 0.1198
$R1$ / $wR2$ (all data)	0.0510 / 0.1224	0.1803 / 0.2453	0.0719 / 0.1624	0.2448 / 0.1901
CCDC №	1965211	1965210	1965209	1980659

According to XRD data in the molecule of **7**, the N–O distances are 1.247(11)–1.291(7) Å. Both N–H moieties form H-bonds with O atoms of nitroxides (O36 and O10) and of corresponding nitroso groups (Figure S1).

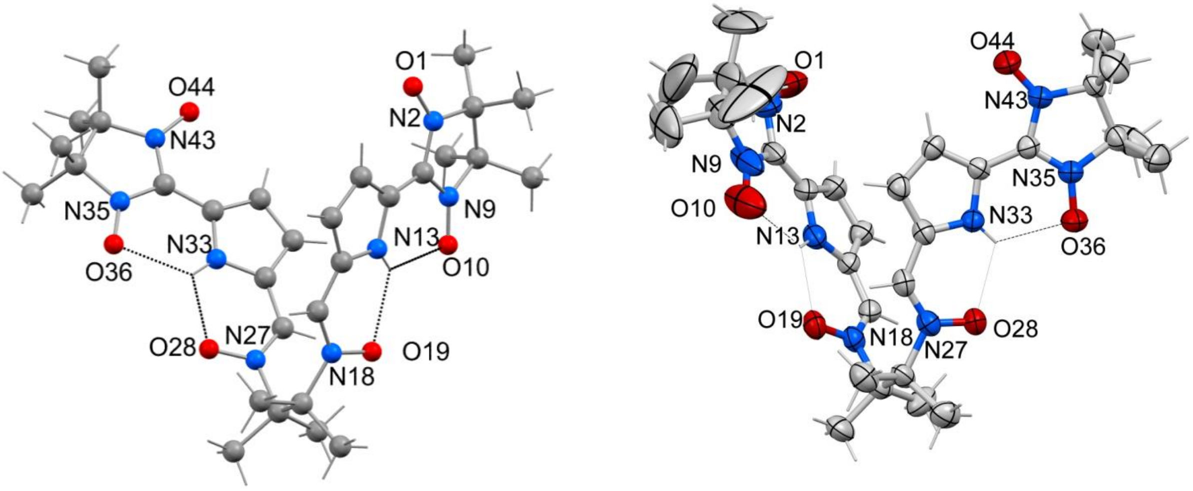

Figure S1. Molecular structure of **7** (intramolecular H-bonds are shown by dotted lines).

Quantum-chemical calculations

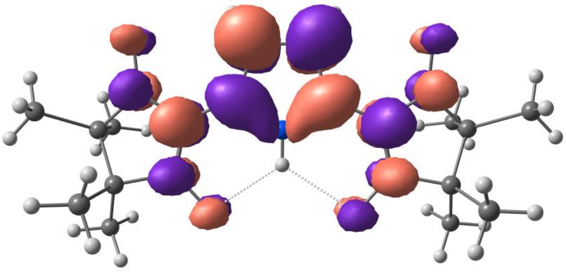<br>106: n = 0.0490, E = 9.4607 eV	
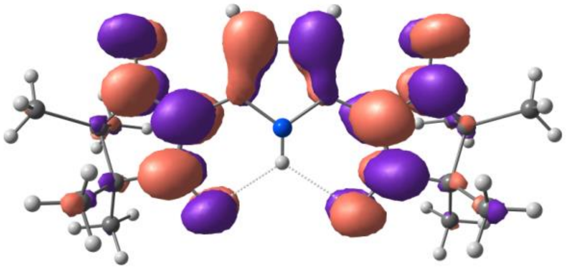<br>104: n = 0.1149, E = 5.2137 eV	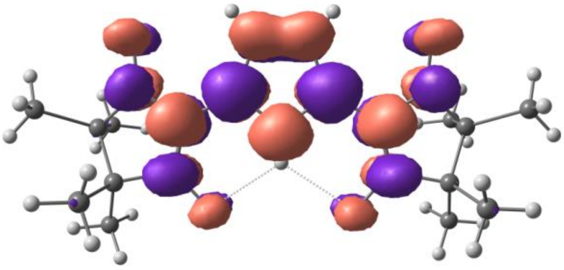<br>105: n = 0.0578, E = 8.8511 eV
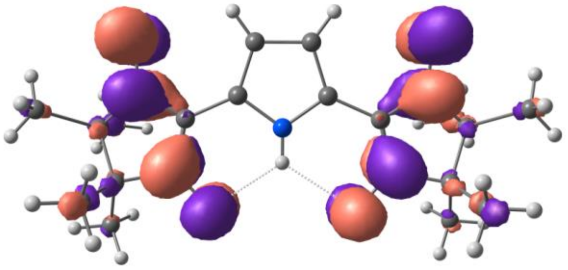<br>102: n = 1.0031, E = –3.3634 eV	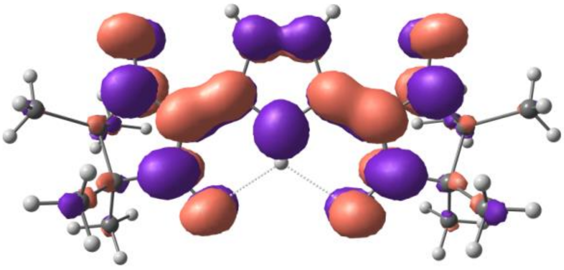<br>103: n = 0.1692, E = 2.6959 eV
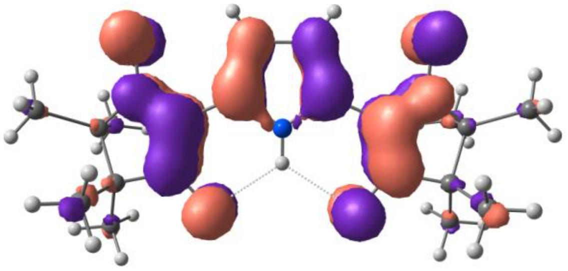<br>100: n = 1.8502, E = –7.9458 eV	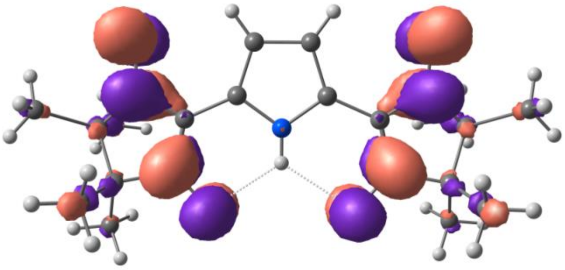<br>101: n = 1.0060, E = –3.3699 eV
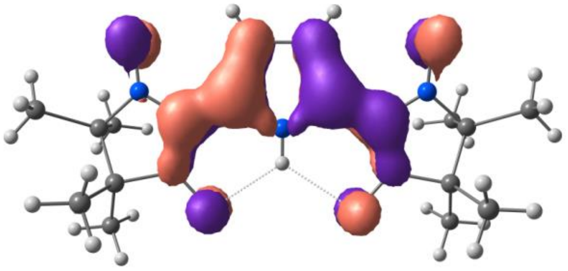<br>98: n = 1.9430, E = –10.9540 eV	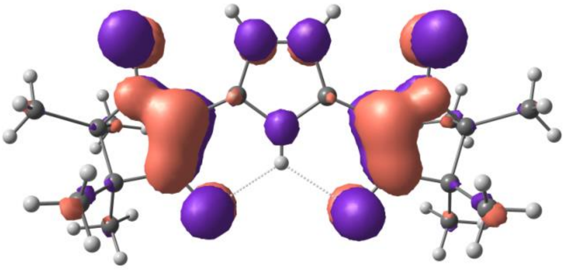<br>99: n = 1.8972, E = –10.4208 eV

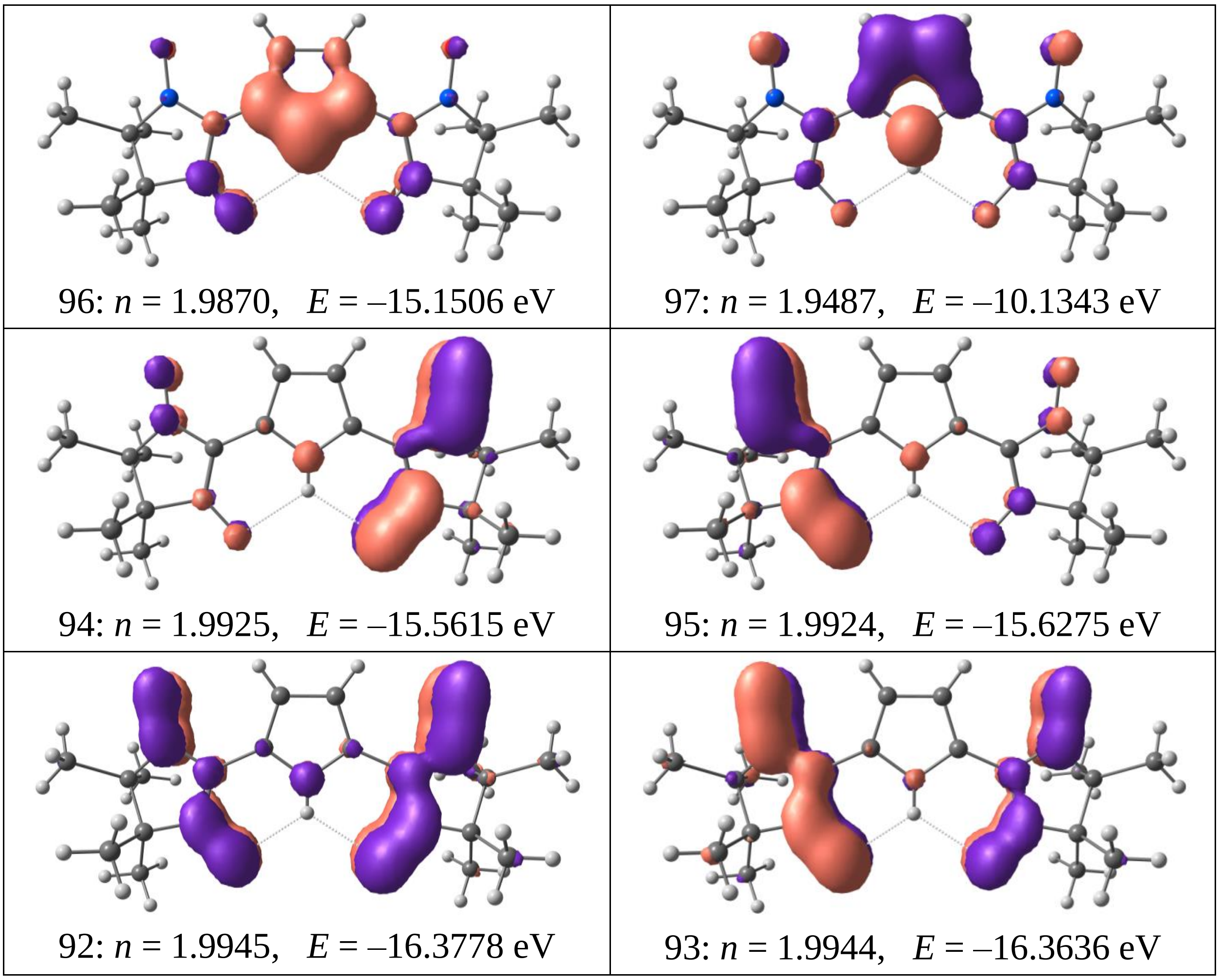

Figure S2. CAS(20,15) MOs obtained for **1**, occupation numbers *n* and energies *E* are taken from the SA-CASSCF calculation. CAS(16,13) includes orbitals 94, 95, 97–106, CAS(10,10) – orbitals 97–106, CAS(6,6) – orbitals 99–104, CAS(2,2) – orbitals 101, 102.

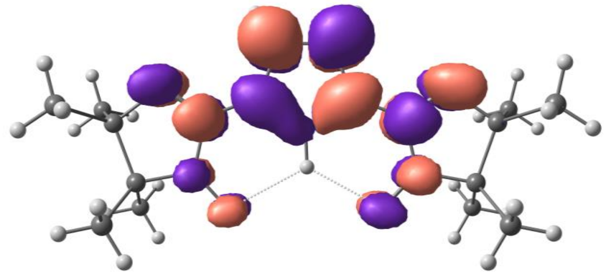

98: n = 0.0471, E = 10.1518 eV

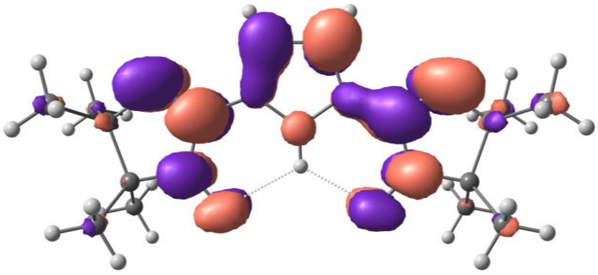

96: n = 0.0875, E = 6.7146 eV

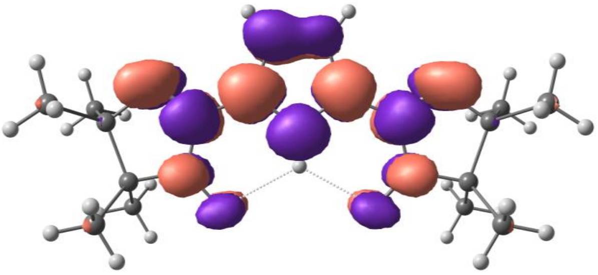

97: n = 0.0528, E = 10.0760 eV

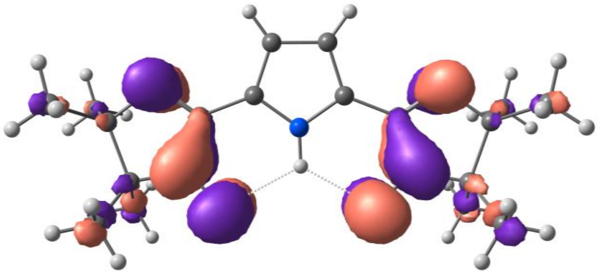

94: n = 1.0012, E = –3.2045 eV

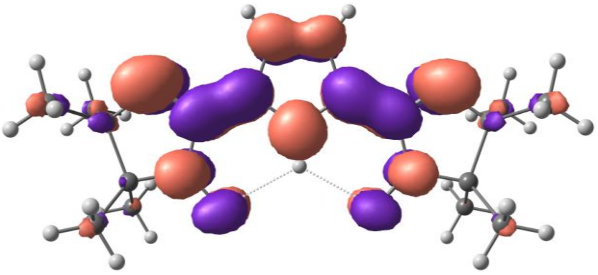

95: n = 0.1302, E = 3.8621 eV

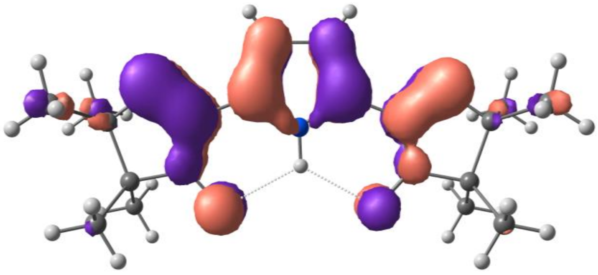

92: n = 1.8810, E = –8.4501 eV

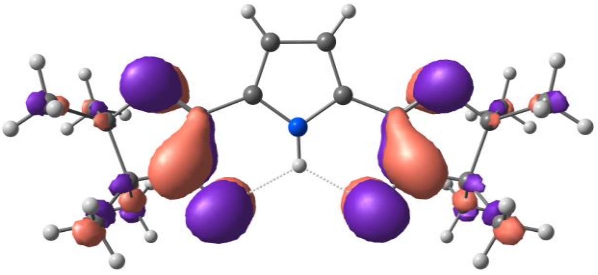

93: n = 1.0076, E = –3.2580 eV

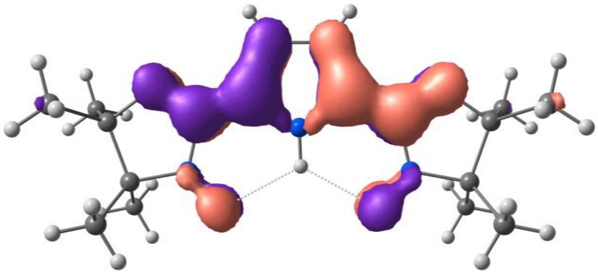

90: n = 1.9463, E = –11.6688 eV

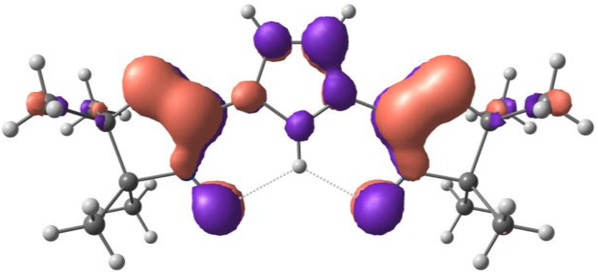

91: n = 1.9172, E = –11.4427 eV

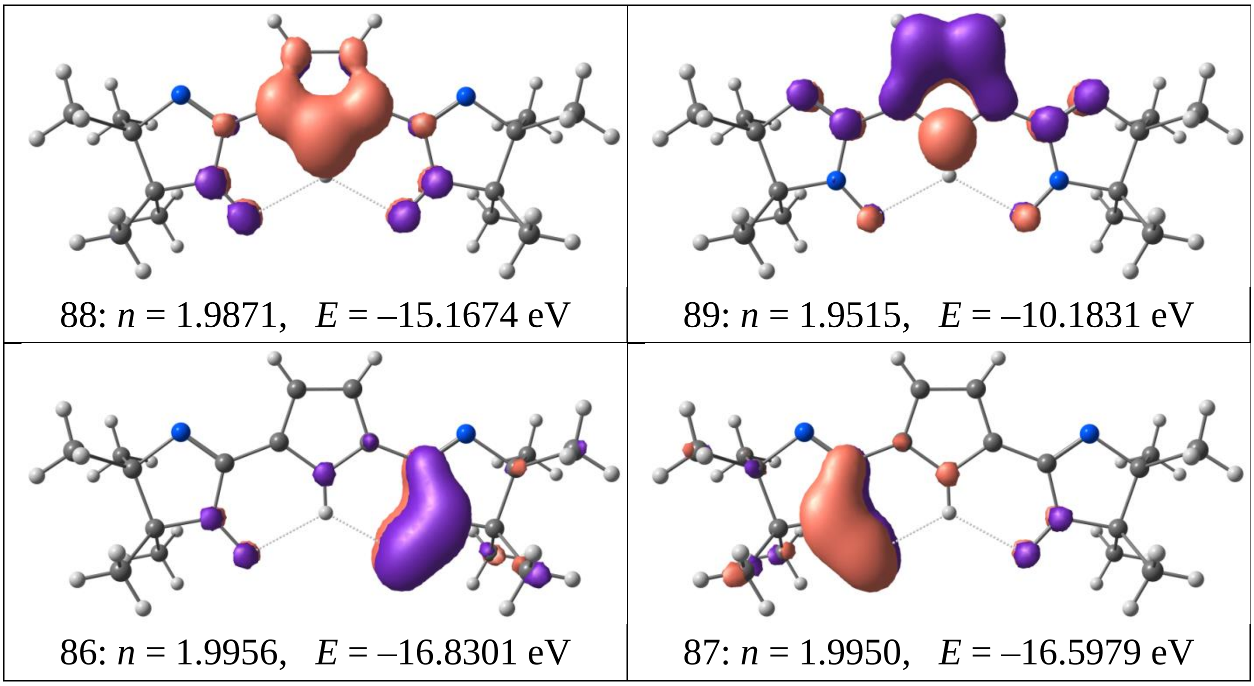

Figure S3. CAS(16,13) MOs obtained for **2**, occupation numbers n and energies E are taken from the SA-CASSCF calculation. CAS(10,10) includes orbitals 89–98, CAS(6,6) – orbitals 91–96, CAS(2,2) – orbitals 93, 94.

CW ESR Spectroscopy

Although diradicals have structures similar to *o*-terphenyl, their solubility in it is still rather low, which leads to glass cracking and deposition of compact phases at crack surface resulting in a narrow signal of compact phases appearing in the spectra in the region g=2 and overlapping the characteristic diradical signal. To distinguish the signal from a diluted solid solution of diradical in bulk glass and from compact phases of diradical the samples were sequentially diluted 2x with neat *o*-terphenyl. This was done by opening the ESR sample tube, melting its contents on a water bath, removing half of the melt by aspiration, and pouring additional neat *o*-terphenyl to obtain the original melt volume. Then the tube was again sealed, frozen and transferred to ESR resonator. In practice such sample preparation procedure after maximum two dilutions resulted in nearly complete elimination of the compact phase signal at the expense of significant decrease of the target signal. The figures below show spectra obtained for several sequential dilutions demonstrating the disappearance of the compact phase signal and separation of the sought signal from glassy diradical solution. This procedure produced the spectra for two symmetric diradicals **1** and **2**.

The non-symmetric diradical **9** with two different radical moieties had a much better solubility in glassy *o*-terphenyl, with spectra practically not changing with dilution. Nevertheless, its spectrum also contains a narrow intense line in the center. Spectrum modelling demonstrated that in this case the diradical has a rather substantial anisotropy parameter, with the ratio E/D = 0.226, which is rather rare and close to the maximum possible value of 1/3. The usual reason for this is a close to orthogonal mutual geometry of the π-orbitals of two upaired electrons (in this case clos to orthogonal mutual orientation of two radical moieties), which also likely explains the much higher solubility of **9** in *o*-terphenyl glass in comparison to nearly coplanar **1** and **2**.

For all three diradicals the spectra in molten glass have also been obtained (Figs. S9, S14, S20), which serve to illustrate the exceptional stability of these systems. Although they look "solution-like" and feature a certain resolved structure, they cannot be faithfully simulated, as they correspond to the situation of slow, probably anisotropic rotation and belong to pairs of exchange-coupled radicals in the case of intermediate exchange, the most difficult case to model that requires exceptional quality of experimental spectra not possible with melts. The only reasonable estimate that can be done with them is to pick the limiting contribution of non-interacting radicals, which is always present in such spectra, and estimate the nitrogen hyperfine couplings. Such an estimate produces 2N×0.76 mT for the NN moiety of diradical **1** and 1N×0.93 mT plus 1N×0.43 mT for the IN moiety of diradical **2**.

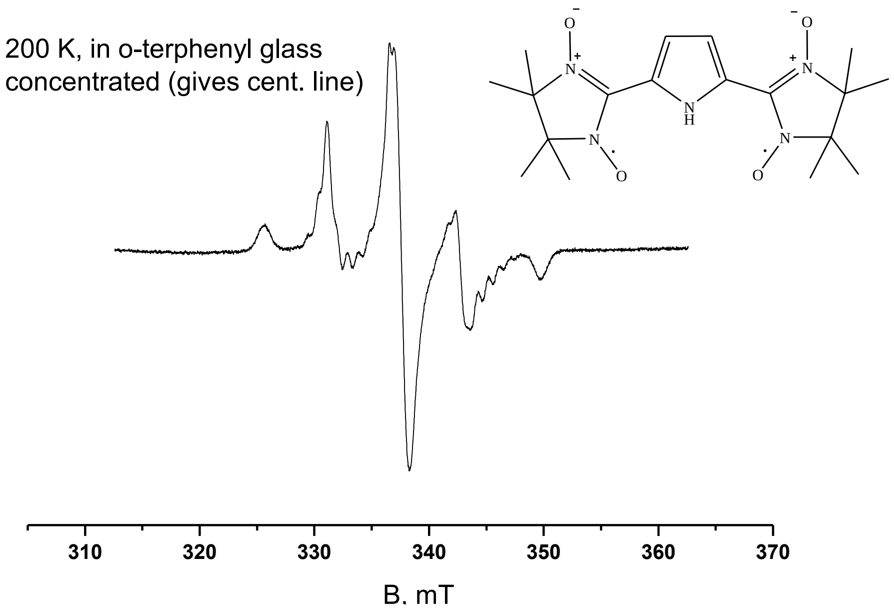

Figure S4. ESR spectrum of glassy sample at a high concentration of **1**, the region of g=2, featuring a characteristic four-line diradical spectrum overlapped with an additional line of compact phases at the center. MW power 2 mW.

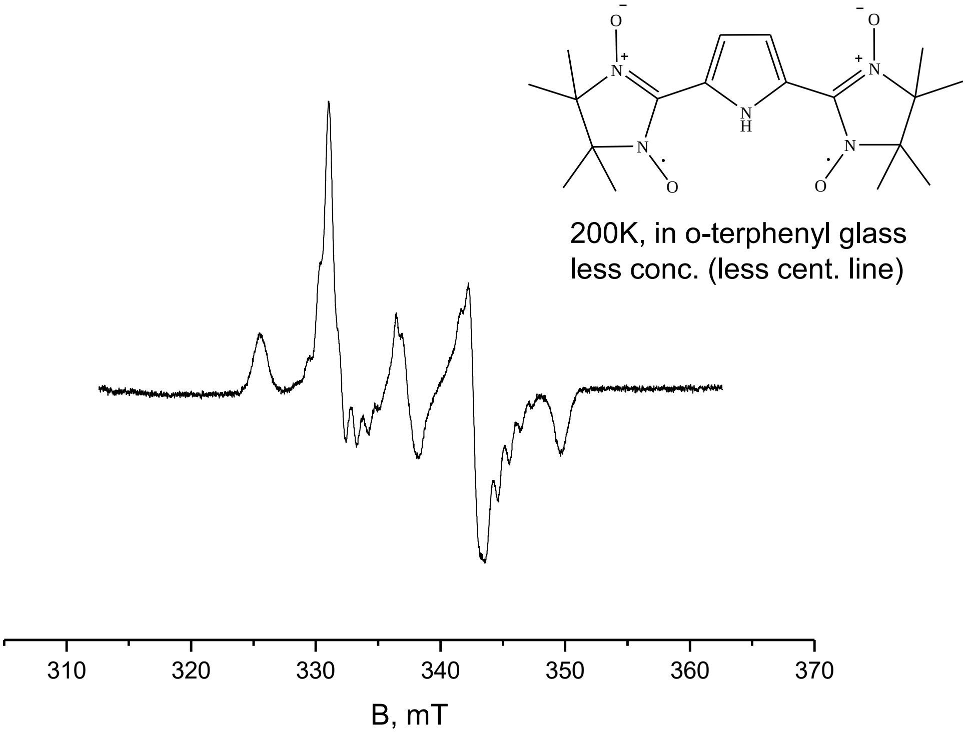

Figure S5. ESR spectrum of **1** in glass, a diluted sample from the preceding figure. The entire signal has decreased in intensity, but the interfering compact phase line dropped more than the diradical spectrum. Region of g=2. MW power 2 mW.

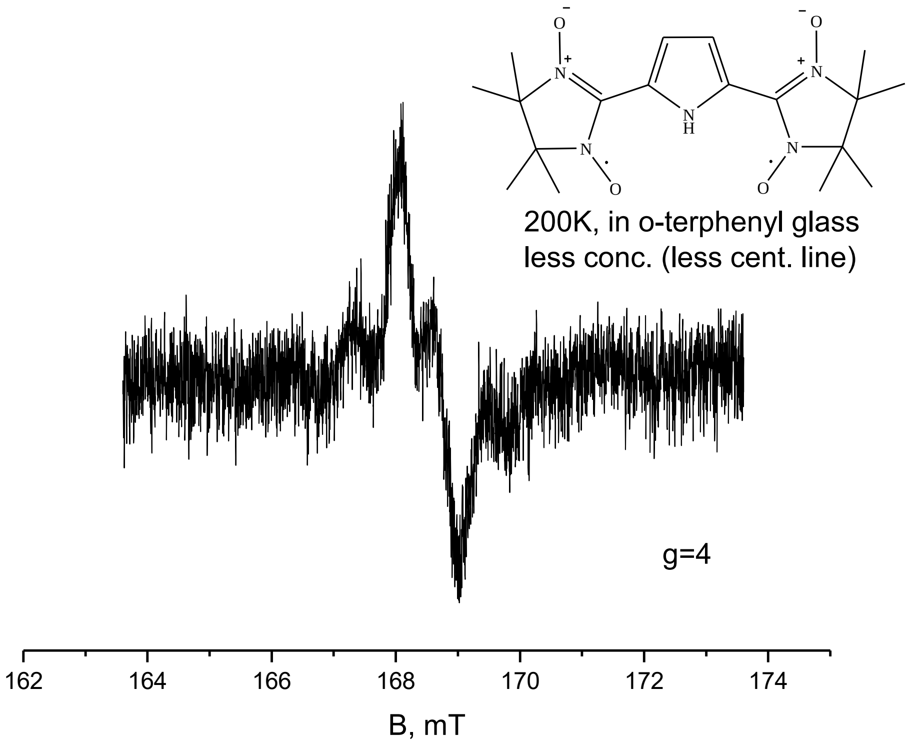

Figure S6. ESR spectrum of **1** in glass, the same diluted sample as in the preceding figure. Region g=4, the “half field line”, MW power 200 mW (practically undetectable at a lower MW power).

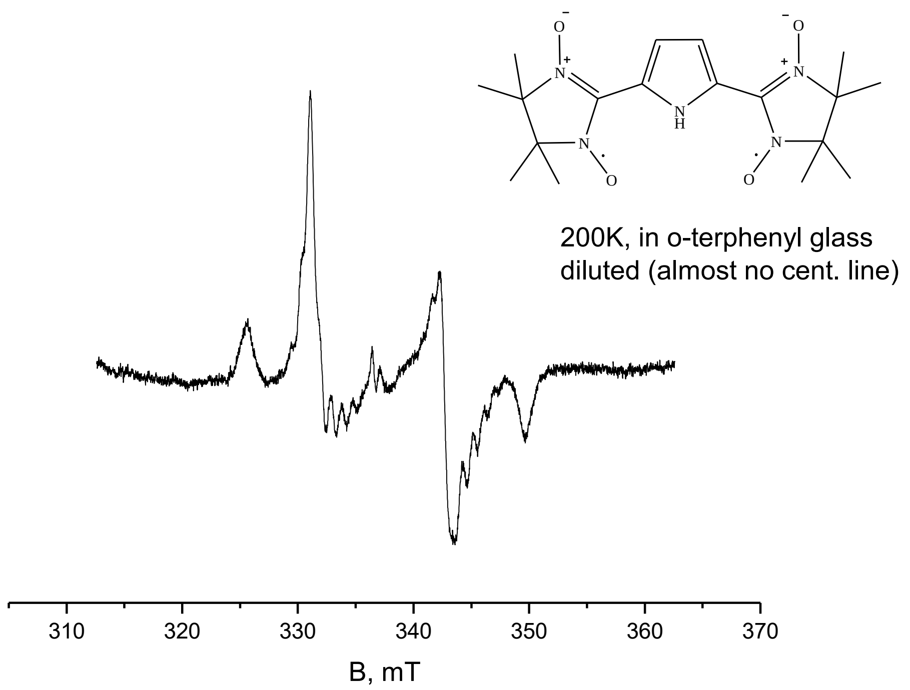

Figure S7. ESR spectrum of **1** in glass, next dilution of the sample from the preceding figure, spectrum acquisition time 6 hrs. The target signal is very weak, but the interfering compact phase line has been nearly completely eliminated with acceptable quality of the final spectrum. Region of g=2. MW power 2 mW.

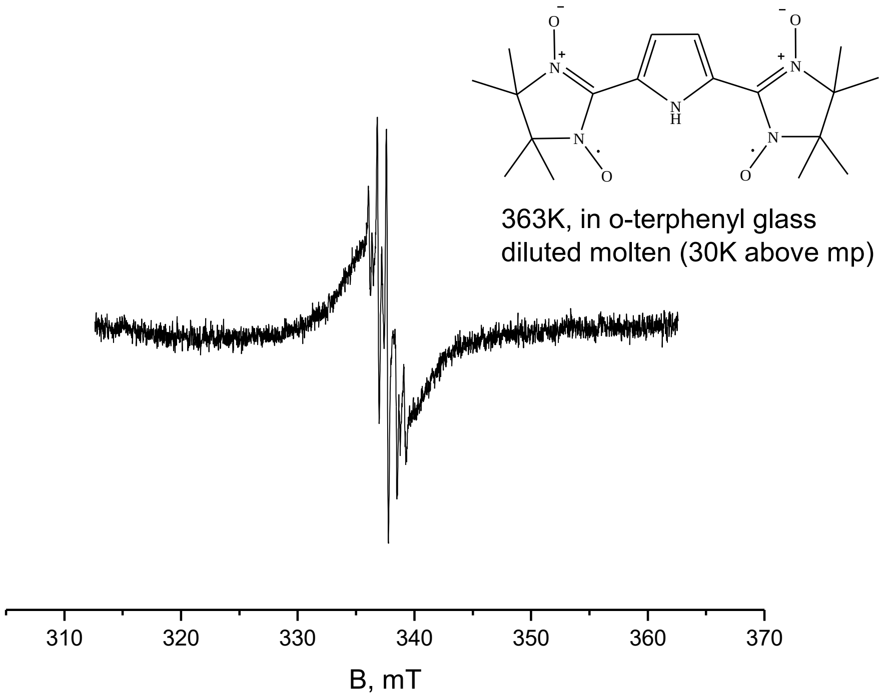

Figure S8. ESR spectrum from a molten sample of **1** in *o*-terphenyl obtained by warming the sample from the previous figure to 30 degrees above the glass melting point directly in the resonator (the diradical proved to be rather stable), which produced a solution-like spectrum. Region of g=2. MW power 2 mW.

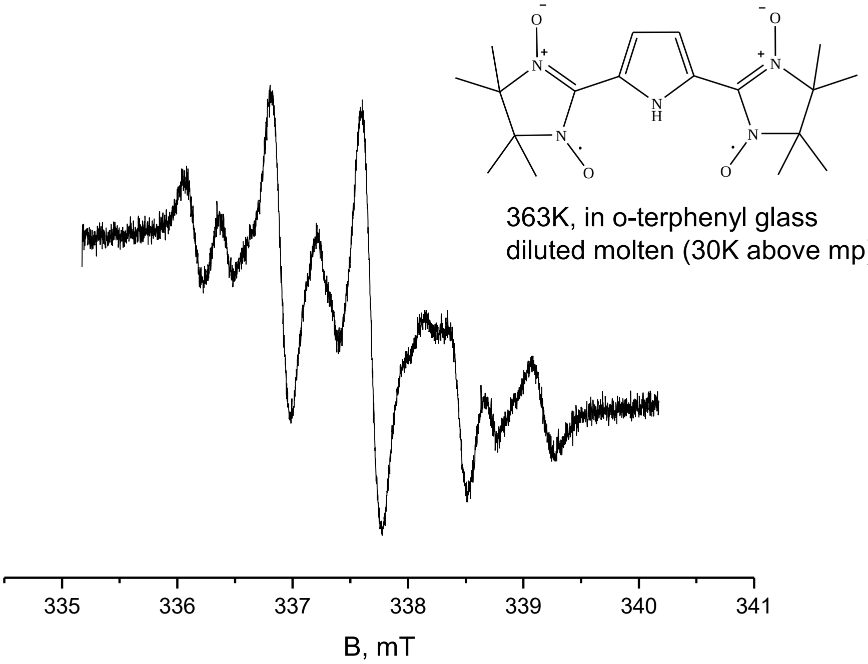

Figure S9. Expanded view of the ESR spectrum from molten sample of **1** in *o*-terphenyl from the previous figure, having a shape expected for a solution spectrum of a diradical, while this sample spent a day at a temperature of +60 – +90 degrees (molten *o*-terphenyl) without any adverse effect for the diradical. Region of g = 2. MW power 2 mW.

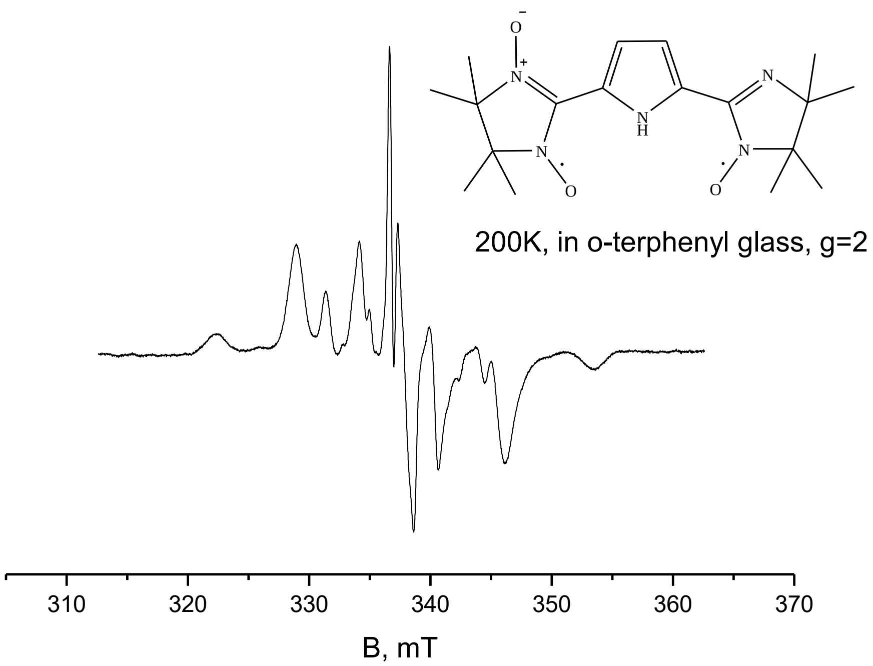

Figure S10. ESR spectrum of diradical **9** in *o*-terphenyl glass, the region of g = 2, MW power 2 mW. Despite the presence of narrow lines in the center this is a purely diradical spectrum (see main text for details).

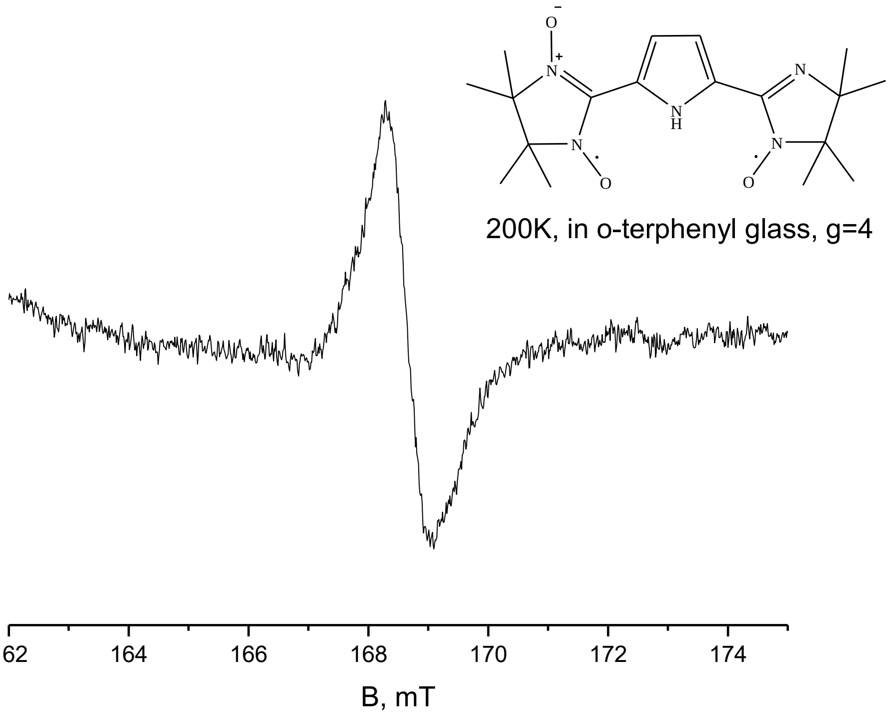

Figure S11. ESR spectrum of **9** in glass, diluted sample. Region g = 4, the “half field line”, MW power 200 mW (practically undetectable at a lower MW power).

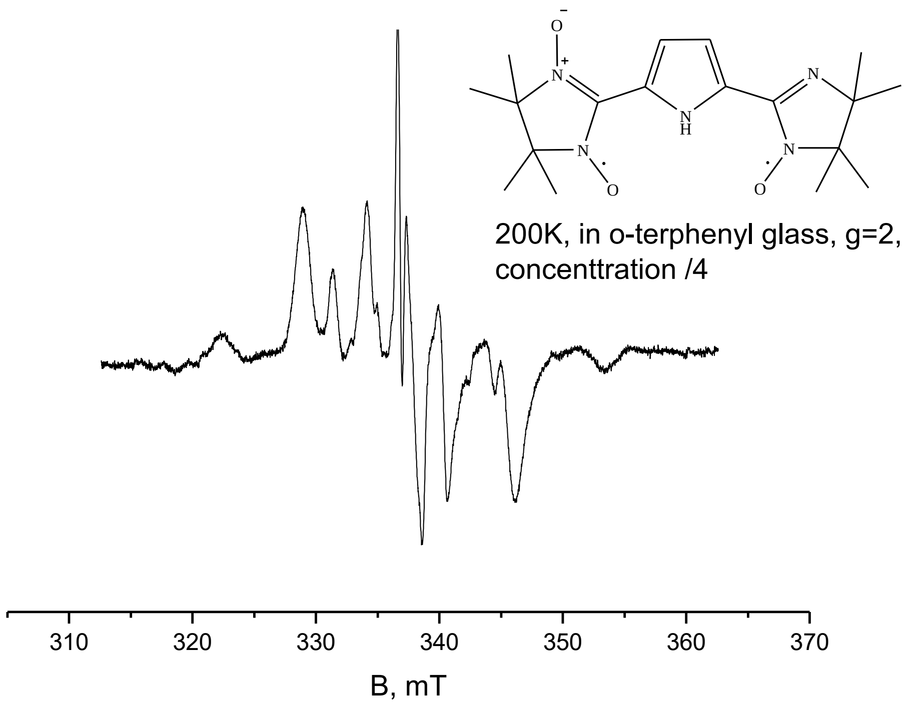

Figure S12. ESR spectrum of **9** in glass, two successive dilutions of the sample from the preceding figure, acquisition time 6 hrs. The target signal is very weak, but the spectrum shape has practically not changed, in stark contrast with **1**. Region of g = 2. MW power 2 mW.

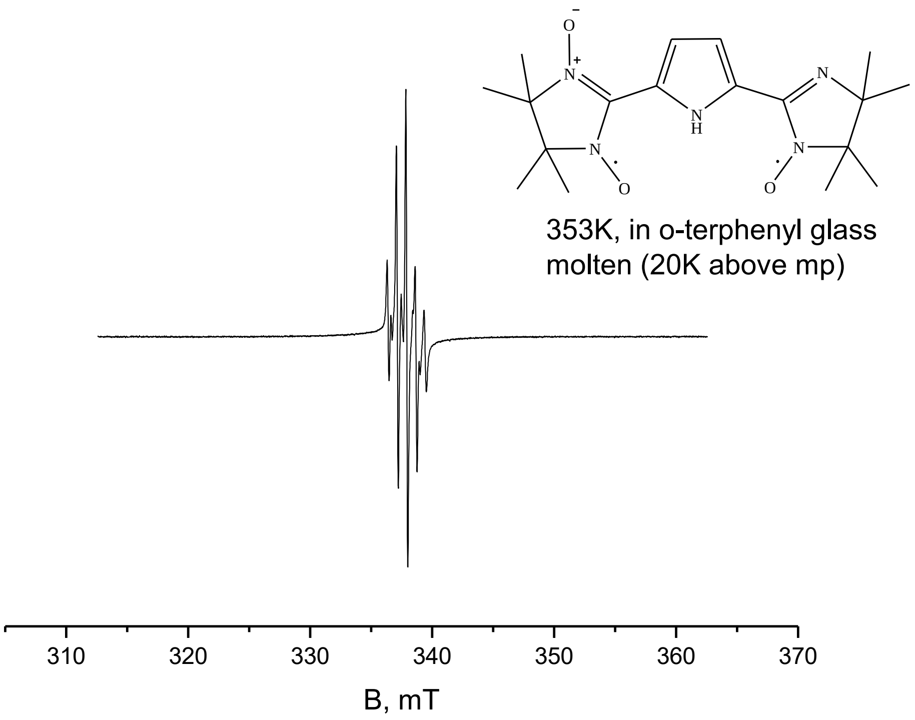

Figure S13. ESR spectrum from a molten sample of **9** in *o*-terphenyl obtained by warming the sample from the previous figure to 20 degrees above the glass melting point directly in the resonator (the diradical proved to be rather stable), which produced a solution-like spectrum. Region of g = 2. MW power 2 mW.

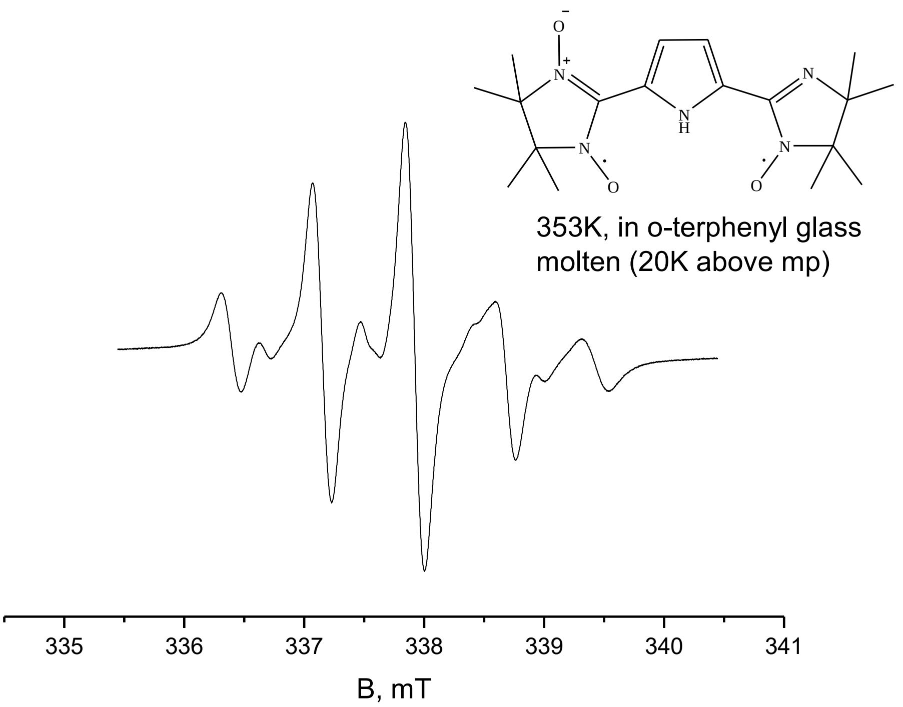

Figure S14. Expanded view of the ESR spectrum from molten sample of **9** in *o*-terphenyl from the previous figure, having a shape expected for a solution spectrum of a diradical. Region of g = 2. MW power 2 mW.

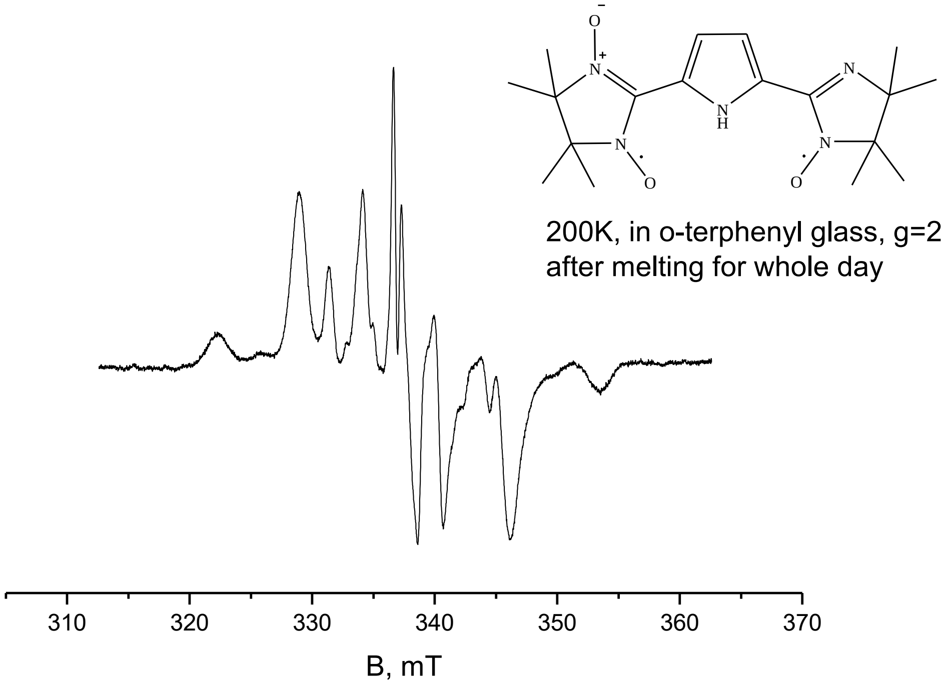

Figure S15. ESR spectrum for the glassy sample of **9** from the previous figure. This spectrum was obtained from the sample that had been measured for a whole day in molten *o*-terphenyl and then was cooled down back to 200K, to obtain the same spectrum as for the original sample, i.e., the diradical is stable to such temperature cycling. Region of g = 2. MW power 2 mW.

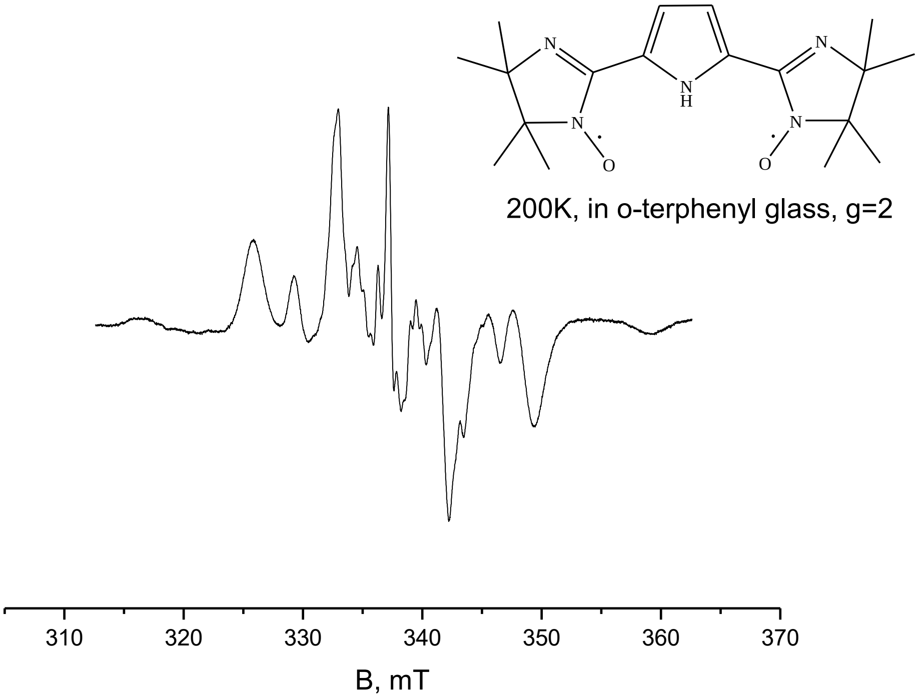

Figure S16. ESR spectrum of glassy sample at a high concentration of **2**, the region of g = 2, again featuring a characteristic four-line diradical spectrum overlapped with an additional line of compact phases at the center. MW power 2 mW.

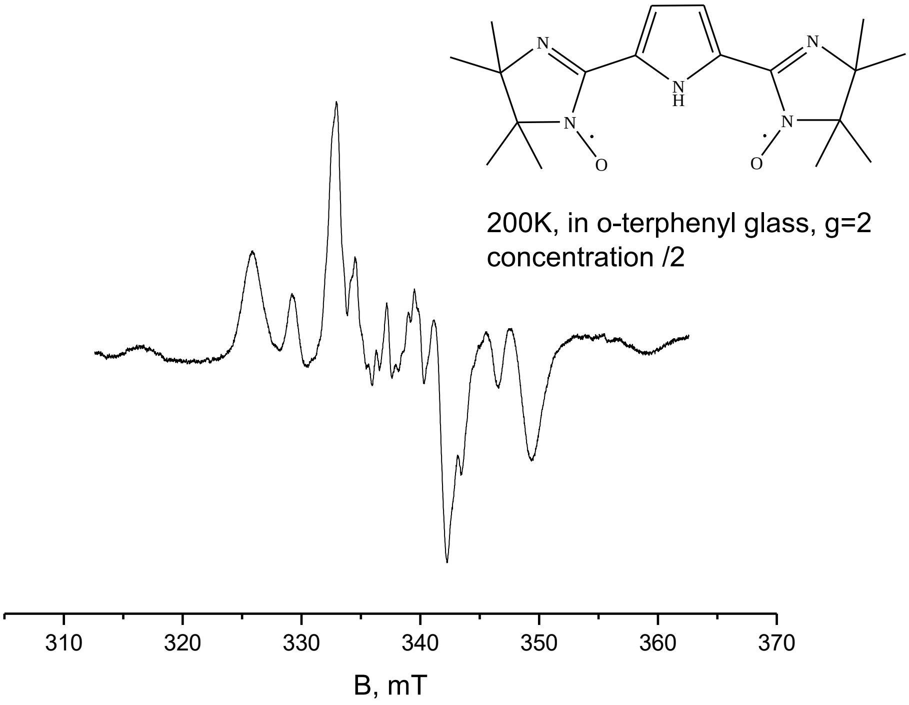

Figure S17. ESR spectrum of **2** in glass, a diluted sample from the preceding figure. The entire signal has decreased in intensity, but the interfering compact phase line dropped more than the diradical spectrum. Region of g = 2. MW power 2 mW.

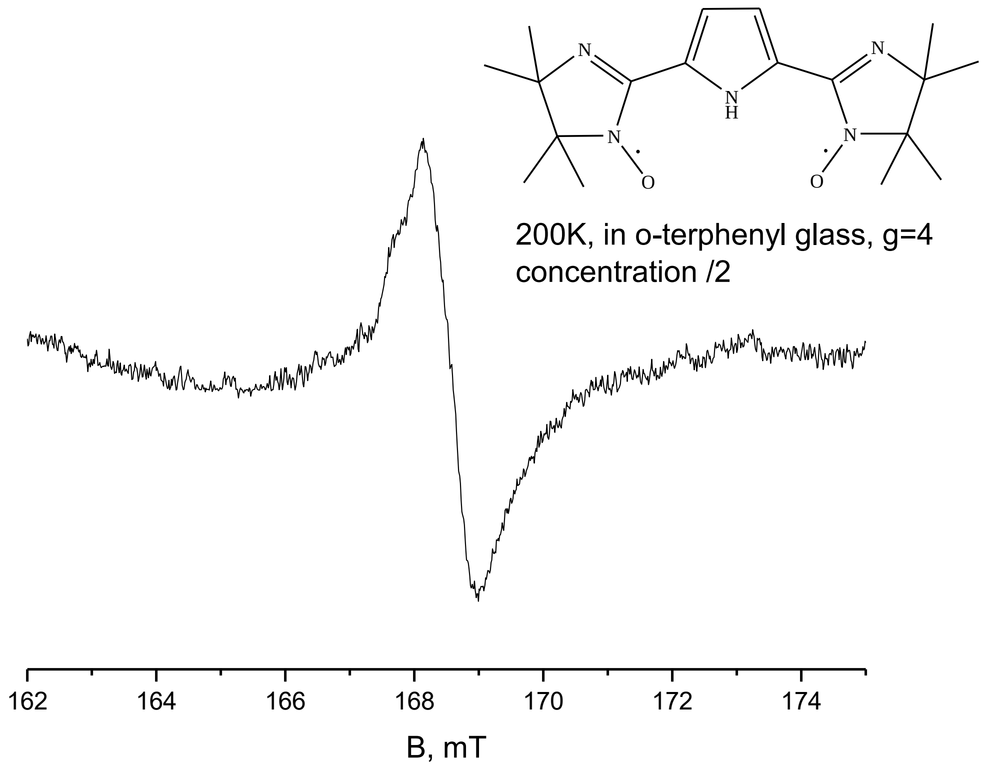

«

Figure S18. ESR spectrum of **2** in glass, diluted sample. Region g = 4, the “half field line”, MW power 200 mW (practically undetectable at a lower MW power).

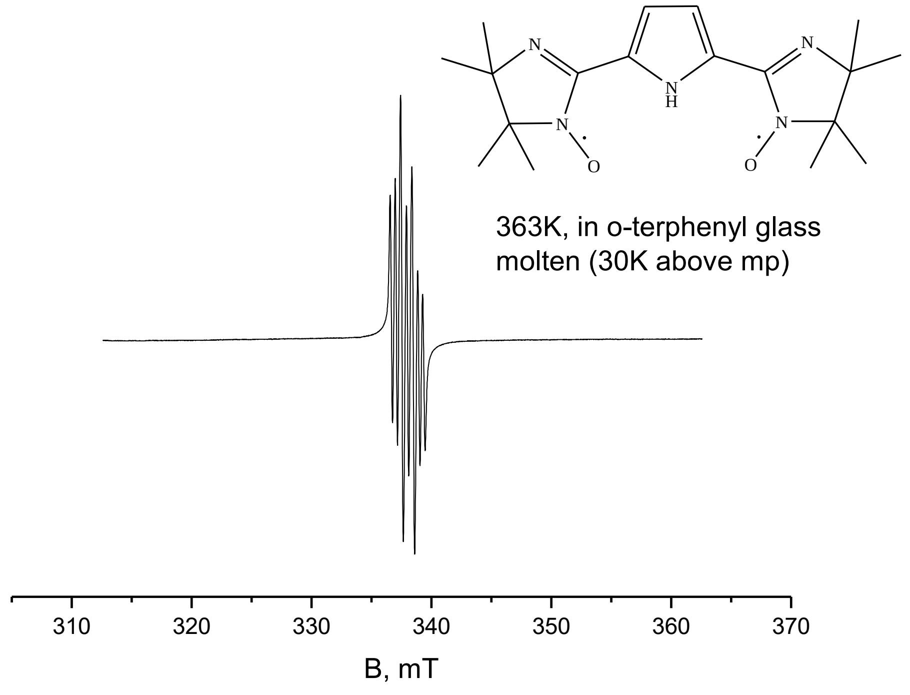

Figure S19. ESR spectrum from a molten sample of **2** in *o*-terphenyl obtained by warming the sample from the previous figure to 30 degrees above the glass melting point directly in the resonator (the diradical proved to be rather stable), which produced a solution-like spectrum. Region of g = 2. MW power 2 mW.

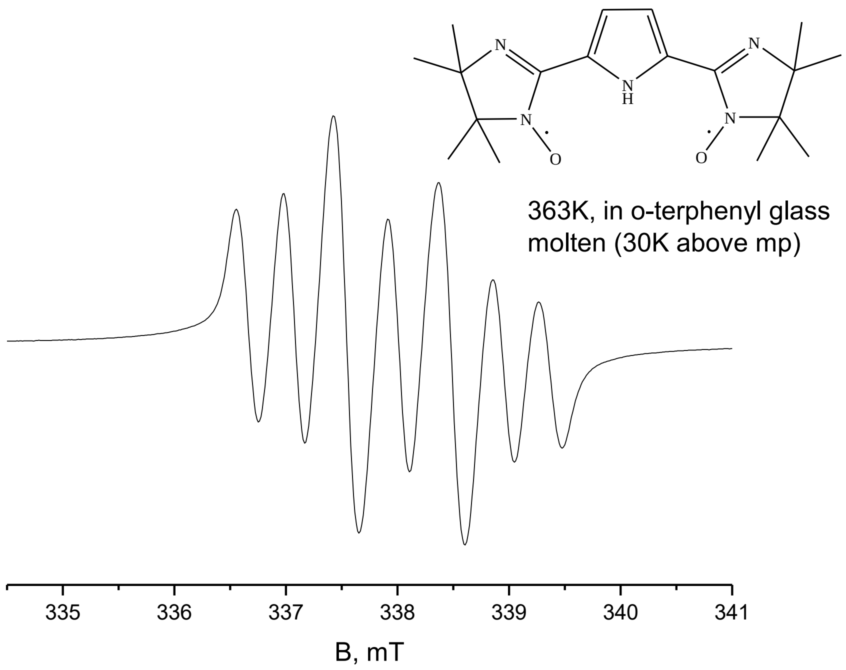

Figure S20. Expanded view of the ESR spectrum from molten sample of **2** in *o*-terphenyl from the previous figure, having a shape expected for a solution spectrum of a diradical, while this sample spent a day at a temperature of +60 – +90 degrees (molten *o*-terphenyl) without any adverse effect for the diradical. Region of g = 2. MW power 2 mW.
